# Mental health special consideration in Victorian tertiary education applications: geographical, demographic, and socioeconomic inequities in uptake

**DOI:** 10.3389/fpsyt.2026.1908088

**Published:** 2026-09-15

**Authors:** Shu Mei Teo, Kate Filia, Kirra Greaves, David G. Baker, Mengmeng Wang, Daniel Z. Q. Gan, Teresa Tjia, Matthew Rudd, Paula Soon, Gavin Peter, Vivienne Browne, Sue Cotton, Gina Chinnery, Eóin Killackey, Caroline Gao

**Affiliations:** 1 Centre for Youth Mental Health, The University of Melbourne, Parkville, VIC, Australia; 2 Orygen, The National Centre of Excellence in Youth Mental Health, Parkville, VIC, Australia; 3 Victorian Tertiary Admissions Centre (VTAC), Melbourne, VIC, Australia; 4 School of Psychological Sciences, Faculty of Medicine, Nursing and Health Sciences, Monash University, Clayton, VIC, Australia; 5 Turner Institute for Brain and Mental Health, Monash University, Clayton, VIC, Australia; 6 School of Public Health and Preventive Medicine, Monash University, Melbourne, VIC, Australia

**Keywords:** educational equity, mental health service utilisation, special entry access scheme, tertiary education access, youth mental health

## Abstract

**Introduction:**

Mental ill-health can disrupt young people’s education and future outcomes. In Victoria, Australia, the Special Entry Access Scheme (SEAS) enables tertiary applicants to declare circumstances, including mental health conditions, that may have affected their educational achievement, yet application patterns have never been comprehensively characterised.

**Methods:**

We analysed de-identified Victorian Tertiary Admissions Centre (VTAC) data from 43,127 Victorian Year-12 applicants in the 2021/22 admissions cycle, examining mental health-related SEAS rates across individuals and Statistical Area Level 3 (SA3) regions. Area-level rates were compared with Census-reported mental health conditions prevalence and acute mental health service use among 17–18-year-olds. Relative risk regression, Spearman correlations, and tertile-based concordance analyses examined demographic, socioeconomic, and geographic factors associated with uptake.

**Results:**

Fifteen percent of applicants disclosed a mental health condition via SEAS. Female applicants were nearly twice as likely as males to apply (RR = 1.92), non-binary applicants more than three times as likely (RR = 3.47), applicants from English-speaking backgrounds twice as likely (RR = 2.01), and those with comorbid learning disability more than three times as likely (RR = 3.47). Financial hardship was associated with higher SEAS uptake (RR = 1.52), yet area-level socioeconomic advantage was also positively associated with disclosure rates (RR = 1.08 per decile), revealing a paradoxical inverse equity pattern. Geographic analysis identified substantial discordance between SEAS application rates and population-level need indicators.

**Discussion:**

SEAS provides a unique opportunity to support young people whose educational pathways have been disrupted or shaped by disadvantage, particularly, but not limited to mental ill-health. At a population level, mental health-related SEAS uptake is not determined by underlying need alone. It is also shaped by wider social and structural factors that influence young people’s ability to recognise, document, and disclose mental health difficulties and their educational impacts. Cultural, geographic, and socioeconomic barriers can exclude many who would benefit, highlighting opportunities to strengthen equitable access to the scheme. Proactive school-based navigation support, targeted outreach to culturally and linguistically diverse communities, and sustained institutional support beyond the point of admission are needed to ensure SEAS fulfils its potential as an equitable access mechanism for young people with mental ill-health.

## Introduction

Mental ill-health is highly prevalent among young people, representing a major threat to their wellbeing, educational participation, and long-term life outcomes. Almost two in five young people aged 16–24 years experience a mental disorder in a 12-month period (1). Recent increases in psychological distress and mental ill-health among adolescents and young adults have been widely reported in Australia and many other countries (2). Adolescence and early adulthood is the peak period for the onset of mental ill-health, with 60–75% of all diagnoses occurring before age 25 (3). When mental ill-health arises during these formative years, they can set in motion a cascade of compounding disadvantage, disrupting education engagement, narrowing employment opportunities, and contributing to social exclusion. These processes undermine recovery and entrench existing inequalities in a vicious cycle (4). Ensuring that young people experiencing mental ill-health have equitable access to educational opportunities is therefore essential for supporting their recovery and future success (5).

To address potential educational inequity, Australian states and territories have implemented special entry access schemes administered through state-based Tertiary Admissions Centres (TACs), to support disadvantaged students to access tertiary education, though these vary in structure and eligibility criteria. In Victoria, this is achieved through the Special Entry Access Scheme (SEAS), a targeted pathway administered by the Victorian Tertiary Admissions Centre (VTAC). This scheme enables applicants to declare circumstances, including pre-existing mental ill-health, that may have adversely affected young people’s educational achievement. SEAS encompasses four non-mutually-exclusive categories covering personal and location factors, financial hardship, disability or medical conditions, as well as difficult life circumstances, each requiring supporting documentations from relevant professionals and independent referees. The scheme operates across most universities and a substantial number of TAFE (providers of vocational education and training) and independent providers across Victoria.

Approximately 70% of Victorian Year 12 students engage with the VTAC admissions process annually (6). By capturing education-related disadvantage (including mental health conditions), SEAS data offers a valuable epidemiological lens on the prevalence of mental ill-health among young people transitioning to tertiary study.

Evidence from longitudinal data suggests that rates of mental ill-health among tertiary students have increased in recent years (7). However, little is known about how this burden is reflected in SEAS applications, particularly the proportion of Year 12 applicants citing mental health as a basis for special consideration, and how this varies by location. This gap matters because variation in mental health-related SEAS applications likely reflects not only underlying differences in prevalence, but also other structural barriers, including differential awareness of SEAS processes, variability in mental health literacy, and access to diagnostic services and the clinical documentation required to support a claim. If left unaddressed, such upstream disparities risk reproducing, rather than mitigating, the very social and health inequities SEAS was designed to redress.

Despite its potential utility for health and education policy, this novel data source has never been fully characterised. A strategic partnership project between VTAC and Orygen was therefore established to support translating data for mental health policy (6). Building on this foundation, the present study integrates SEAS application data with population-level mental health prevalence and service use data to examine who applies for mental health special consideration in Victoria, and who may not be reaching this pathway despite need. Specifically, we aimed to: (i) characterise the demographic, socioeconomic, and geographic profile of Victorian Year-12 applicants who apply for mental health-related SEAS consideration; (ii) examine how mental health applications intersect with other forms of disadvantage captured through SEAS, including financial hardship, difficult life circumstances, and comorbid health conditions; (iii) explore whether geographic disparities in mental health-related SEAS application rates exist; and (iv) assess the degree to which SEAS application patterns align with known population-based mental health prevalence and service utilisation data. Understanding these patterns is essential for ensuring that special consideration pathways reach all young people experiencing mental ill-health, not only those with the resources, awareness, or support to navigate the application process.

## Methods

### Data source and context

VTAC administers SEAS alongside standard tertiary applications for prospective students seeking entry to Victorian universities, vocational institutions, and independent tertiary providers. SEAS encompasses four non-mutually-exclusive categories: (1) Personal information: including Aboriginal and/or Torres Strait Islander heritage, residence in regional or remote areas, non-English speaking background, or first-generation university attendance, (2) Financial disadvantage: including receipt of government income support or other indicators of financial hardship, (3) Medical condition or disability: encompassing physical health conditions, mental health conditions, and learning disabilities, (4) Difficult circumstances: including experiences such as homelessness, family disruption, refugee or asylum seeker status, caring responsibilities, impacts from natural disasters, or disruptions of remote learning. All SEAS applications require supporting documentation from relevant professionals (e.g., medical practitioners, school counsellors, social workers) and/or independent referees to substantiate claims. This study focused specifically on applications citing mental health conditions within the medical condition/disability category.

### VTAC data

This study analysed de-identified, VTAC application data for the 2021/22 admissions cycle. It is important to note that the 2021/22 admissions cycle was atypical in two respects. First, it followed Victoria’s prolonged COVID-19 lockdowns, which totalled 262 days across 2020–2021 and substantially disrupted the secondary schooling, mental health, and social development of this cohort. Second, SEAS Category 4 (difficult circumstances) was temporary expanded during this period to include a COVID-specific option allowing students to declare pandemic-related educational disruption as a basis for special consideration. As a result, rates of Category 4 applications, and co-occurrence between Category 4 and other SEAS categories, in this dataset are substantially higher than would be expected in a typical admissions cycle and should not be taken as indicative of baseline patterns. The main analysis was restricted to Victorian residents aged 18 and under to focus on the primary Year-12 cohort transitioning directly from secondary to tertiary education (cohort hereafter referred to as ‘Year-12’). Applicants over 18 were excluded as mature-age entry typically involves different pathways and circumstances that may confound patterns in mental health SEAS applications during this critical developmental transition. Interstate applicants were excluded because comparisons with Victoria-specific mental health service utilisation data and demographic characteristics coded to Victorian geographic boundaries required a Victorian-resident sample. Data included self-reported demographic information including age category, gender (male, female, non-binary), and residential location coded to Statistical Area Level 2 (SA2) boundaries. Due to small numbers at the SA2 level, residential locations were aggregated to Statistical Area Level 3 (SA3) regions for area-based analyses. SA3 regions represent functional regional towns and cities or clusters of suburbs around urban commercial hubs with populations typically between 30,000 and 130,000 people (8).

### Census data

Mental health prevalence data were obtained from the 2021 Australian Bureau of Statistics (ABS) Census of Population and Housing, which collected data on self-reported long-term health conditions. Census data were extracted using the ABS Table Builder platform. Two custom tables were constructed at the SA3 geographic boundary, filtered to respondents aged 17–18 years to align with the Year-12 cohort of interest.

The first table extracted total population counts for 17–18-year-olds by SA3, to be used as population denominators (see *Population Denominators* section). The second table cross-tabulated SA3 by self-reported mental health condition status, specifically the Census variable *Whether has Mental Health Condition (including Depression or Anxiety)*, yielding counts of individuals who reported having a mental health condition, those who did not, and those with a not-stated response.

Area-level mental health prevalence was calculated for each SA3 as the proportion of 17–18-year-olds who reported having a mental health condition out of all respondents with a stated response, excluding not-stated cases from both the numerator and denominator. These SA3-level prevalence estimates were then used as a contextual benchmark against which SEAS mental health application rates were compared. Importantly, the Census mental health variable was derived from a question asking respondents whether they had been told by a doctor or nurse that they have one or more selected long-term health conditions, with mental health conditions (including depression or anxiety) among the conditions listed. This framing means that Census-based prevalence estimates are likely to underestimate true population prevalence, as they capture only those who have consulted a health professional, received a diagnosis, and accurately recalled and disclosed this at the time of enumeration.

### Service use data

Mental health-related service use data were obtained from three Victorian administrative datasets: the Victorian Emergency Minimum Dataset (VEMD), the Victorian Admitted Episode Database (VAED), and the Victorian Ambulance Clinical Information System (VACIS), managed by Ambulance Victoria. These datasets capture emergency department presentations, hospital admissions, and ambulance attendances, respectively. Access to service use data was facilitated through the Centre for Victorian Data Linkage (CVDL) (9).

Mental health-related episodes were identified within each dataset using a mental health flag. In the VAED and VEMD, episodes were identified using the derived mental health need indicator, which flags individuals who have received, been referred to, or have a potential need for psychiatric, psychological, or mental health-related treatments or services. In VACIS, episodes were identified using the mental health flag, which indicates that the patient meets Ambulance Victoria’s mental health specifications criteria. For each dataset, the number of unique individuals aged 17–18 years with at least one mental health-related service contact during 2021 was counted within each SA3, forming the numerator. These counts were divided by the SA3-level Census-based population denominators described below and multiplied by 1,000 to yield rates of mental health service use per 1,000 youth aged 17–18 years.

### Population denominators

Since Year-12 students span two birth cohorts (typically turning 17 or 18 during their final year), the 17–18-year-olds Census counts were halved to approximate a single Year-12 cohort within each SA3 for use as population denominators in VTAC rate calculations. This approach assumes population size is broadly stable across adjacent single-year ages (17 and 18 years) within each SA3. Census-reported prevalence estimates and service use were not subject to this adjustment, as both numerator and denominator were drawn from the same age group. One SA3 with a halved Census-based population denominator of fewer than 150 youth (i.e., fewer than 300 17–18-year-olds in the 2021 Census; Loddon - Elmore) was excluded from all area-level analyses due to unreliable rate estimation, leaving 65 SA3s for these analyses.

### Area-level socioeconomic advantage

Area-level socioeconomic advantage was measured using the Index of Relative Socio-economic Advantage and Disadvantage (IRSAD), one of four Socio-Economic Indexes for Areas (SEIFA) produced by the Australian Bureau of Statistics from 2021 Census data (8). IRSAD summarises a range of Census variables reflecting both advantage (e.g., high income, skilled occupation) and disadvantage (e.g., low income, unemployment) into a single score for each SA2, with higher scores indicating greater relative advantage. IRSAD scores were assigned at the SA2 level based on applicants’ residential address and expressed as within-state percentile rank and decile. SA2 regions that did not receive an IRSAD score due to insufficient resident population was imputed using the mean score of SA2 regions within the same SA3.

### Statistical methods

All analyses were conducted using R version 4.5.2 (10). Descriptive analyses characterised the demographic, socioeconomic, and educational profiles of the Victorian Year-12 cohort, stratified by mental health SEAS application status. Mental health SEAS application rates were calculated at the area level as both the proportion of VTAC applicants within each SA3 disclosing a mental health condition (crude rate) and the number of mental health SEAS applications per 1,000 estimated population (population rate). Area-level rates were examined by remoteness classification using the Australian Statistical Geography Standard Remoteness Structure. Representativeness of the VTAC cohort relative to the broader Victorian Year-12 population was assessed by comparing cohort size and demographic composition against 2021 ABS Census estimates for 17–18-year-olds.

### Relative risk regression

To examine individual-level predictors of mental health-related SEAS application, relative risk regression models were fitted using individual VTAC application records as the unit of analysis (N = 43,127). Relative Risks (RRs) were estimated using Poisson regression with a log link function and SA3-clustered robust sandwich variance estimation, which provides valid relative risk estimates for binary outcomes without the convergence limitations of log-binomial models (11), while accounting for the geographical nesting of applicants within SA3 regions which may share broader socioeconomic and geographical characteristics that could induce correlated residuals within region. Univariate models were first fitted for each predictor individually. A multivariable model was then fitted including all predictors simultaneously: gender (male, female, non-binary), home language (English vs. other), Aboriginal and/or Torres Strait Islander status, Greater Melbourne residence, learning disability, physical condition, special consideration for financial hardship, first-in-family university attendance, and area-level socioeconomic advantage (IRSAD decile ranking). As a sensitivity analysis, the models were re-fitted excluding mental health SEAS applications without recorded supporting evidence, comparing applicants with a documented, supported mental health SEAS application against applicants who did not apply for mental health SEAS.

### Geospatial comparisons with population-level mental health indicators.

To examine relationships between SEAS applications and population-level mental health indicators at area level, Spearman rank correlations were computed between all pairs of measures across the 65 SA3 regions. Variables included in the correlation analysis were: VTAC mental health SEAS application rates (SEAS crude rate), VTAC mental health SEAS applications per 1,000 population (SEAS population rate), census-reported mental health conditions (census rate), ambulance mental health callouts per 1,000 youth aged 17–18 (ambulance rate), emergency department mental health presentations per 1,000 youth aged 17–18 (ED rate), hospital mental health admissions per 1,000 youth aged 17–18 (hospitalisation rate), and any acute mental health service use per 1,000 youth aged 17–18, capturing youth who had at least one ambulance callout, ED presentation, or hospital admission (Any MH service rate). Correlations were examined both overall and stratified by area-level socioeconomic tertile (low, medium, high IRSAD) to assess whether associations varied by socioeconomic context.

To complement the continuous correlation analysis, a tertile-based concordance analysis was conducted to assess geographic alignment between SEAS application rates and population need indicators. Each SA3 was independently classified into low, medium, or high tertiles based on its SEAS crude rate, Census-reported mental health prevalence, and composite acute service utilisation rate (any MH service contact per 1,000). Tertile cut-points were derived empirically from the observed distribution of each measure across SA3s. Concordance was defined as an SA3 falling within the same tertile category on both SEAS application rates and the comparator indicator. Discordance was classified as higher (SEAS tertile exceeding the comparator indicator tertile) or lower (SEAS tertile below the comparator indicator tertile).

## Results

### Overview of VTAC applications

This study focused on the 43,127 VTAC applications received from Year-12 applicants residing in Victoria for the 2021/22 admissions cycle. An additional 19,528 applications were received from older Victorian applicants (n=11,475) and non-Victorian applicants (n=8,053; 6,246 Year-12; 1,807 older); these groups were excluded from the primary analysis given differences in application context and circumstances but are described briefly in **Supplementary Table 1**.

The Victorian Year-12 cohort was predominantly female (56.1%), Australian-born (80.5%), English-speaking (82.7%), and residing in Greater Melbourne (83.2%). Fifteen percent (n=6,347) applied for mental health SEAS consideration. The full demographic, socioeconomic, and educational characteristics stratified by mental health SEAS application status are presented in **Table 1**.

**Table 1:** Participant characteristics for Year-12 Victorian VTAC applications for the 2021/22 admissions cycle (N = 43,127) by mental health SEAS application.

Characteristic	All applicants (N = 43,127)	Mental health SEAS applicants (n=6,347)	Other applicants (n=36,780)
	Column %	Row %	Row %
Gender			
Male	18706 (43.4%)	1814 (10%)	16892 (90%)
Female	24189 (56.1%)	4445 (18%)	19744 (82%)
Non-binary	232 (0.5%)	88 (38%)	144 (62%)
Country of birth			
Australia	34736 (80.5%)	5527 (16%)	29209 (84%)
Other	8391 (19.5%)	820 (10%)	7571 (90%)
Home language			
English	35663 (82.7%)	5762 (16%)	29901 (84%)
Other	7464 (17.3%)	585 (8%)	6879 (92%)
Aboriginal and/or Torres Strait Islander			
No	42847 (99.4%)	6297 (15%)	36550 (85%)
Yes	280 (0.6%)	50 (18%)	230 (82%)
Number of parents/guardians completed tertiary education			
None	12116 (28.1%)	1428 (12%)	10688 (88%)
One	10614 (24.6%)	1578 (15%)	9036 (85%)
Both	20357 (47.2%)	3332 (16%)	17025 (84%)
First in family attending university			
No	34929 (81.0%)	5137 (15%)	29792 (85%)
Yes	8198 (19.0%)	1210 (15%)	6988 (85%)
IRSAD percentile			
Mean (SD)	59.8 (27.9)	65.2 (27.7)	58.9 (27.8)
Median (Q1, Q3)	62 (39, 86)	72 (43, 89)	62 (38, 83)
Special consideration for financial hardship			
No	36078 (83.7%)	5066 (14%)	31012 (86%)
Yes	7049 (16.3%)	1281 (18%)	5768 (82%)
Special consideration for difficult life or family challenges			
No	12816 (29.7%)	1058 (8%)	11758 (92%)
Yes	30311 (70.3%)	5289 (17%)	25022 (83%)
ATAR categories			
<50	5937 (14.6%)	745 (13%)	5192 (87%)
(50,70]	11567 (28.5%)	1904 (16%)	9663 (84%)
(70,80]	7223 (17.8%)	1176 (16%)	6047 (84%)
(80,90]	7684 (18.9%)	1156 (15%)	6528 (85%)
(90,95]	3967 (9.8%)	579 (15%)	3388 (85%)
>95	4269 (10.5%)	474 (11%)	3795 (89%)
Region			
Greater Melbourne	35891 (83.2%)	5422 (15%)	30469 (85%)
Rest of Victoria	7236 (16.8%)	925 (13%)	6311 (87%)
Physical condition			
No	42640 (98.9%)	6209 (15%)	36431 (85%)
Yes	487 (1.1%)	138 (28%)	349 (72%)
Learning disability			
No	42506 (98.6%)	6004 (14%)	36502 (86%)
Yes	621 (1.4%)	343 (55%)	278 (45%)

The ‘All applicants’ column shows the distribution of characteristics across the entire Victorian Year-12 cohort (column percentages). The ‘Mental Health SEAS applicants’ and ‘Other applicants’ columns show row percentages, indicating the proportion of each demographic group who did or did not apply for mental health SEAS. ATAR: Australian Tertiary Admission Rank, a rank between 0 and 99.95 indicating a student’s position relative to their peers. IRSAD: Index of Relative Socio-economic Advantage and Disadvantage; higher scores indicate greater relative socioeconomic advantage.

### Representativeness of the VTAC cohort

To contextualise the VTAC sample relative to the broader Victorian population of the same age, cohort size and demographic composition were compared against 2021 ABS Census estimates. The Victorian Year 12-aged population denominator was approximated by averaging the Census counts for 17- and 18-year-olds, approximately 71,615 young people. The VTAC Victorian Year-12 cohort of 43,127 therefore represents approximately 60% of this estimated population. Data from the On Track survey indicate that approximately 72% of 2021 Year-12 completers remained in education or training in the following year (12), suggesting that the VTAC cohort plausibly captures most of those who completed secondary school and sought tertiary entry. It should be noted, however, that students completing Unscored VCE or VCE Vocational Major are less likely to apply through VTAC, as these pathways are not oriented toward ATAR-based tertiary entry, and this population may include students with the greatest unmet need.

In terms of demographic composition, females were over-represented in the VTAC cohort relative to Census estimates (56% vs. 49%), reflecting well-documented patterns of higher female participation in tertiary education (13). These comparisons should be interpreted with some caution as VTAC recorded self-reported gender while the Census collected sex.

### Prevalence and geographic distribution across Victorian SA3s

Victorian Year-12 VTAC applications were distributed across 66 SA3s in Victoria. Application rates, calculated across 65 SA3s (see Methods), varied across SA3s (*Mdn* = 550.3 per 1,000 population, I*QR* = 413.8–766.3), reflecting geographical differences in the proportion of young people seeking tertiary education.

Among VTAC applicants, the proportion who applied for mental health SEAS ranged from 8% to 24% across SA3s (*Mdn* = 14%, *IQR* = 11%–17%), indicating considerable geographic variation in mental health disclosure rates among young people seeking tertiary education. When mental health SEAS applications were expressed as a population-based rate, rates had a median of 72.9 (I*QR* = 54.9–123.4) per 1,000 population, reflecting the number of young people in each community seeking both tertiary education and mental health accommodations. **Figure 1** presents the geographic distribution of these rates across Victorian SA3s, revealing substantial spatial variation. Detailed rates for all SA3s are provided in **Supplementary Table 2**.

**Figure 1:**
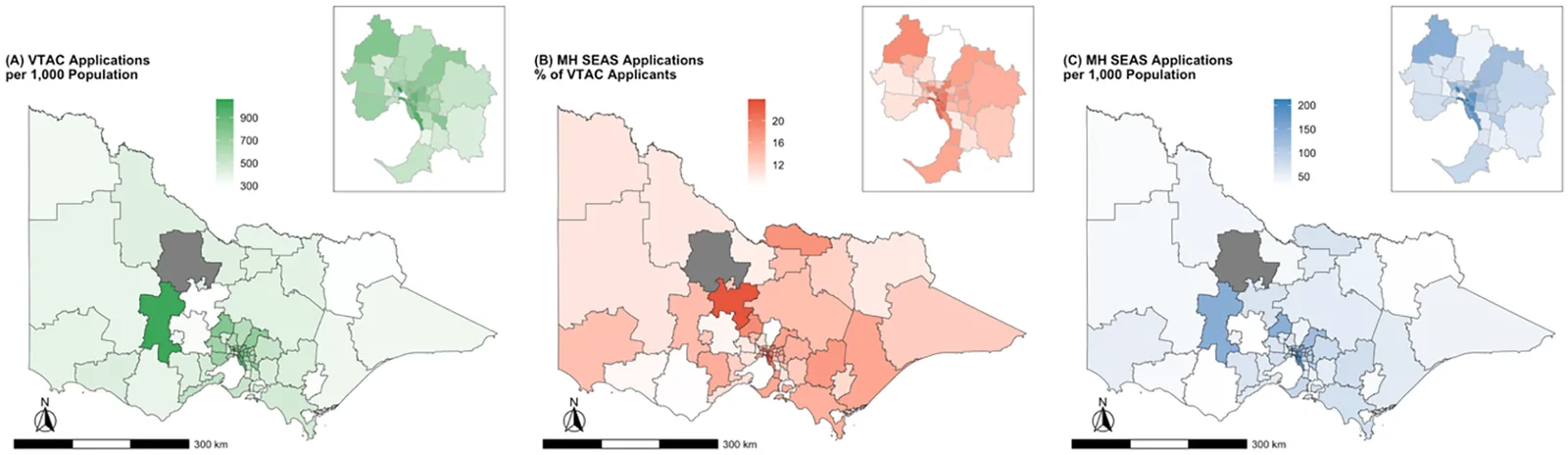
Geographic distribution of VTAC applications and mental health SEAS applications across Victorian SA3 regions, 2021/22. **(A)** Total VTAC applications per 1,000 population; **(B)** Mental health SEAS applications as a proportion of VTAC applicants; **(C)** Mental health SEAS applications per 1,000 population. Population denominators derived from 2021 ABS Census estimates of 17–18-year-olds, halved to approximate a single Year-12 cohort. One SA3 (Loddon–Elmore) was excluded from analysis due to a Year-12 population denominator below 150, resulting in unstable rate estimates, and is shown in grey. Insets show Greater Melbourne.

When examined by remoteness classification, VTAC application rates were highest in major cities (*Mdn* = 697 per 1,000 youth), compared to inner regional (*Mdn* = 413 per 1,000) and outer regional areas (*Mdn* = 385 per 1,000), reflecting higher rates of tertiary education participation in metropolitan areas. Among VTAC applicants, the proportion of mental health SEAS applications was slightly higher in major cities (15.7%) than in inner or outer regional areas (13.7% and 11.5% respectively). Interestingly, this gradient was reversed in Census-reported mental health prevalence, which was slightly higher in regional areas (inner regional: 12%, outer regional: 11%) than major cities (10%). At the population level, these patterns were compounded by lower overall VTAC participation in regional areas, resulting in population-based SEAS application rates more than twice as high in major cities (97.9 per 1,000) than in outer regional areas (43.7 per 1,000).

### Factors associated with mental health SEAS applications

**Table 2** presents univariate and multivariable relative risk regression models examining factors associated with mental health SEAS applications. Unadjusted univariate analyses showed similar patterns to the adjusted models, with effect sizes remaining largely consistent after controlling for covariates. In the fully adjusted model, gender emerged as a strong predictor: mental health SEAS applications were nearly twice as common among female applicants (RR = 1.92, 95% CI 1.82–2.03) compared to males, with 18% of females applying versus 10% of males (**Table 1**). The association was particularly pronounced for non-binary applicants (RR = 3.47, 95% CI 2.84–4.25), among whom 38% applied for mental health consideration.

**Table 2:** Univariate and multivariable relative risk regression models examining factors associated with mental health SEAS applications among Victorian Year-12 VTAC applicants, 2021/22 admissions cycle (N = 43,127).

	Univariate		Multivariable	
Predictor	RR (95% CI)	p-value	RR (95% CI)	p-value
Female (Ref:Male)	1.89 (1.78 – 2.02)	<0.001	1.92 (1.82 – 2.03)	<0.001
Non-binary (Ref:Male)	3.91 (3.24 – 4.73)	<0.001	3.47 (2.84 – 4.25)	<0.001
Learning disability	3.91 (3.56 – 4.30)	<0.001	3.47 (3.14 – 3.84)	<0.001
Home language – English (Ref: Other)	2.06 (1.83 – 2.33)	<0.001	2.01 (1.79 – 2.26)	<0.001
Physical condition	1.95 (1.66 – 2.28)	<0.001	1.78 (1.51 – 2.09)	<0.001
Special consideration for financial hardship	1.29 (1.19 – 1.41)	<0.001	1.52 (1.42 – 1.63)	<0.001
Greater Melbourne (Ref: Rest of Vic)	1.18 (1.04 – 1.34)	0.010	1.07 (0.94 – 1.21)	0.318
IRSAD decile*	1.08 (1.06 – 1.09)	<0.001	1.08 (1.06 – 1.10)	<0.001
Aboriginal and/or Torres Strait Islander	1.22 (0.94 – 1.57)	0.134	1.09 (0.84 – 1.42)	0.495
First family member attending university	1.00 (0.92 – 1.10)	0.937	1.00 (0.94 – 1.07)	0.926

*Higher IRSAD decile ranking indicate greater relative socioeconomic advantage and lower levels of disadvantage within an area.

Comorbidity with other health conditions showed strong associations. Over half (55%) of applicants with learning disabilities applied for mental health SEAS (RR = 3.47, 95% CI 3.14–3.84), as did 28% of those with physical conditions (RR = 1.78, 95% CI 1.51–2.09).

Socioeconomic factors were significant but notably complex. While applicants experiencing financial hardship were 52% more likely to apply for mental health SEAS (RR = 1.52, 95% CI 1.42–1.63; 18% vs 14% application rate), the relationship between area-level socioeconomic advantage and mental health disclosure operated in the opposite direction: each decile increase in area-level IRSAD was associated with an 8% higher rate of mental health applications (RR = 1.08, 95% CI 1.06–1.10), with more advantaged applicants disclosing at higher rates. **Figure 2** further reveals this gradient to be non-linear, with mental health SEAS disclosure rates remaining relatively flat across the lower half of the IRSAD distribution before rising steeply among applicants from the most advantaged areas.

**Figure 2:**
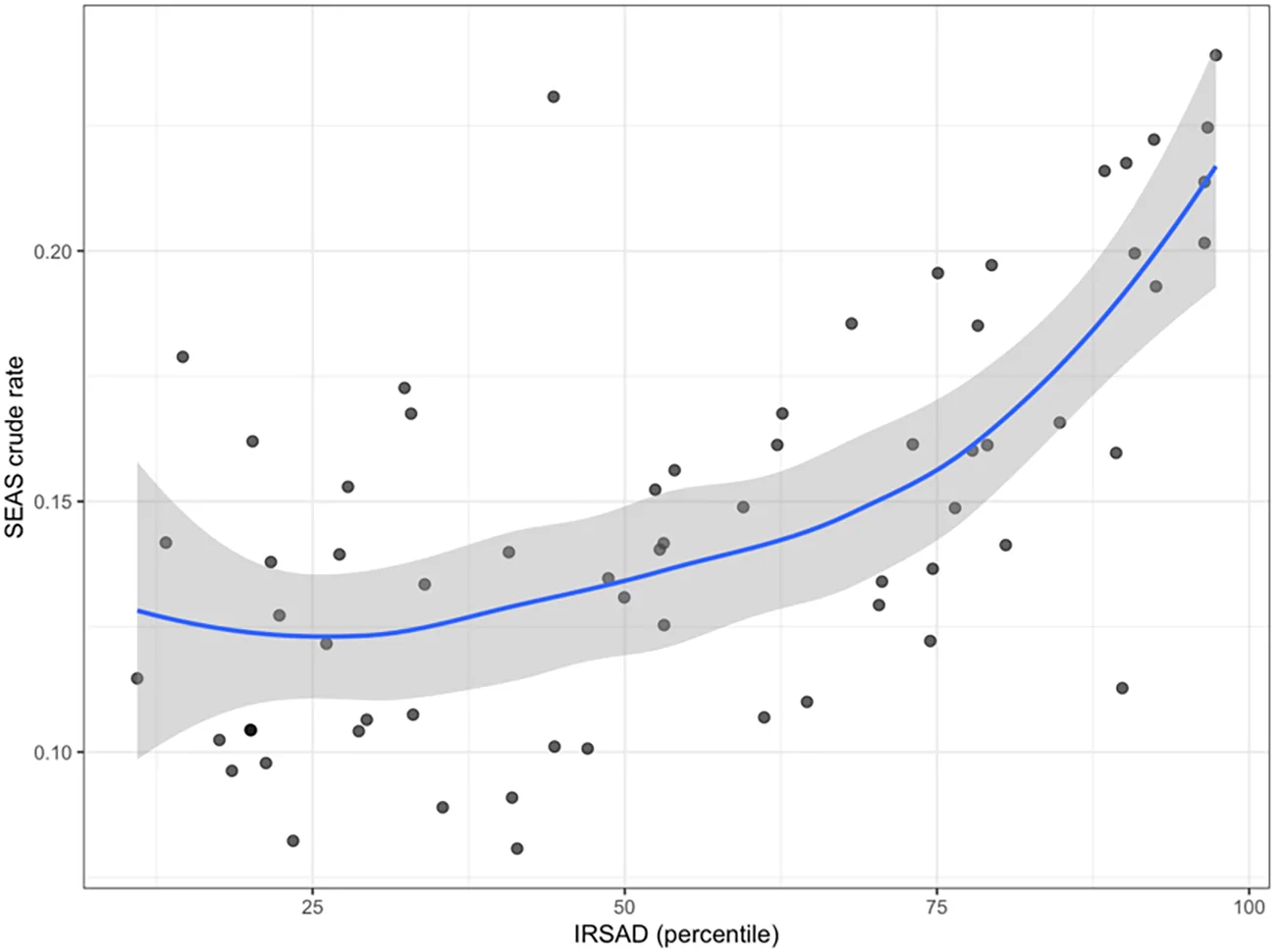
SEAS crude rate against area-level socioeconomic advantage (IRSAD percentile).

Applicants from English speaking backgrounds were twice as likely to apply for mental health SEAS (RR = 2.01, 95% CI 1.79–2.26), with only 16% applying compared to 8% among speakers of other languages. After adjustment, applicants from Greater Melbourne did not differ significantly from those in the rest of Victoria (RR = 1.07, 95% CI 0.94–1.21, p=0.318). Aboriginal and/or Torres Strait Islander status and being a first-generation university student were not significantly associated with mental health SEAS applications.

As a sensitivity analysis, the models were re-fitted excluding mental health SEAS applications without recorded supporting evidence (19% of the mental health SEAS cohort), comparing applicants with a documented, supported mental health SEAS application (n = 5,107) against applicants who did not apply for mental health SEAS (n = 36,780) (**Supplementary Table 3**). All predictors significant in the primary analysis remained significant in this restricted analysis, and both non-significant predictors (Aboriginal and/or Torres Strait Islander status, first-in-family university attendance) remained non-significant. Gender associations were, if anything, stronger under this stricter case definition (female RR 1.92 vs. 2.26; non-binary RR 3.47 vs. 4.08, multivariable estimates).

### Intersectionality: multiple SEAS categories

Most mental health SEAS applicants (89.0%) also applied under at least one other SEAS category, compared to 78.0% of non-mental health applicants.

Difficult circumstances was the most prevalent co-occurring category for both groups, though rates were higher among mental health SEAS applicants (83.3%) compared to non-mental health applicants (68.0%). These high rates across both groups must be interpreted within the context of Victoria’s COVID-19 experience. This cohort completed their final years of secondary education (2020-2021) during Victoria’s prolonged COVID-19 lockdowns, which severely disrupted learning, assessments, and social development for most students.

When examining the specific nature of difficult circumstances, 78.6% of mental health SEAS applicants reported COVID impacts compared to 65.6% of non-mental health applicants. In contrast, only 4.7% of mental health applicants and 2.5% of non-mental health applicants reported non-COVID difficult circumstances only (such as homelessness, family disruption, refugee status, or caring responsibilities).

Personal information considerations were reported by 26.6% of mental health applicants versus 34.0% of non-mental health applicants, representing a lower rate of co-occurrence. Financial hardship was reported by 20.2% versus 15.7%, and other medical conditions or disabilities by 13.7% versus 3.3%.

### Area-level acute mental health service utilisation among Victorian youth

To examine whether mental health SEAS application patterns aligned with objective measures of mental health service use, SA3-level rates of mental health SEAS applications were compared with contemporaneous mental-health service utilisation rates for youth aged 17–18 years in calendar year 2021, corresponding to the year of VTAC application submission.

Across Victorian SA3 regions, mental health-related hospital admission rates for youth aged 17–18 years ranged from 6.0 to 29.1 per 1,000 population (median = 13.6, IQR = [11.2–17.2]). Emergency department presentation rates for mental health concerns ranged from 7.5 to 40.6 per 1,000 population (median = 17.9, IQR = [13.8–22.6]), while mental health-related ambulance attendance rates ranged from 4.8 to 27.0 per 1,000 population (median = 13.6, IQR = [10.5–17.2]). Among youth with at least one acute mental health service contact, rates ranged from 19.3 to 58.8 per 1,000 population. Substantial geographic variation was observed across all three service types, with higher emergency department presentation rates in inner regional areas (median = 21.7 per 1,000) compared to major cities (median = 17.0 per 1,000) and outer regional areas (median = 16.7 per 1,000). A similar pattern was observed for ambulance attendances, while hospital admission rates showed less variation by remoteness, remaining relatively consistent across major cities (median = 14.4 per 1,000), inner regional (median = 12.0 per 1,000), and outer regional areas (median = 11.6 per 1,000).

### Correlations between mental health SEAS application, census-reported mental health conditions, and acute mental health service utilisation

The correlation analysis revealed several notable patterns in the relationships between mental health-related SEAS crude rates, census-reported mental health prevalence, and acute mental health service utilisation across Victorian SA3 regions (**Figure 3**, see **Supplementary Table 4** for SES-tertile-stratified correlations). Strong intercorrelations were observed among acute service utilisation measures. Emergency Department presentations and ambulance attendances were strongly correlated overall (r = 0.69), consistent across all SES tertiles. Hospital admissions showed weaker but significant correlations with ED presentations (r = 0.35) and ambulance attendances (r = 0.34), with these associations most pronounced within high-SES areas (r = 0.72 and r = 0.63 respectively), suggesting greater coherence in acute service use patterns in more advantaged communities.

**Figure 3:**
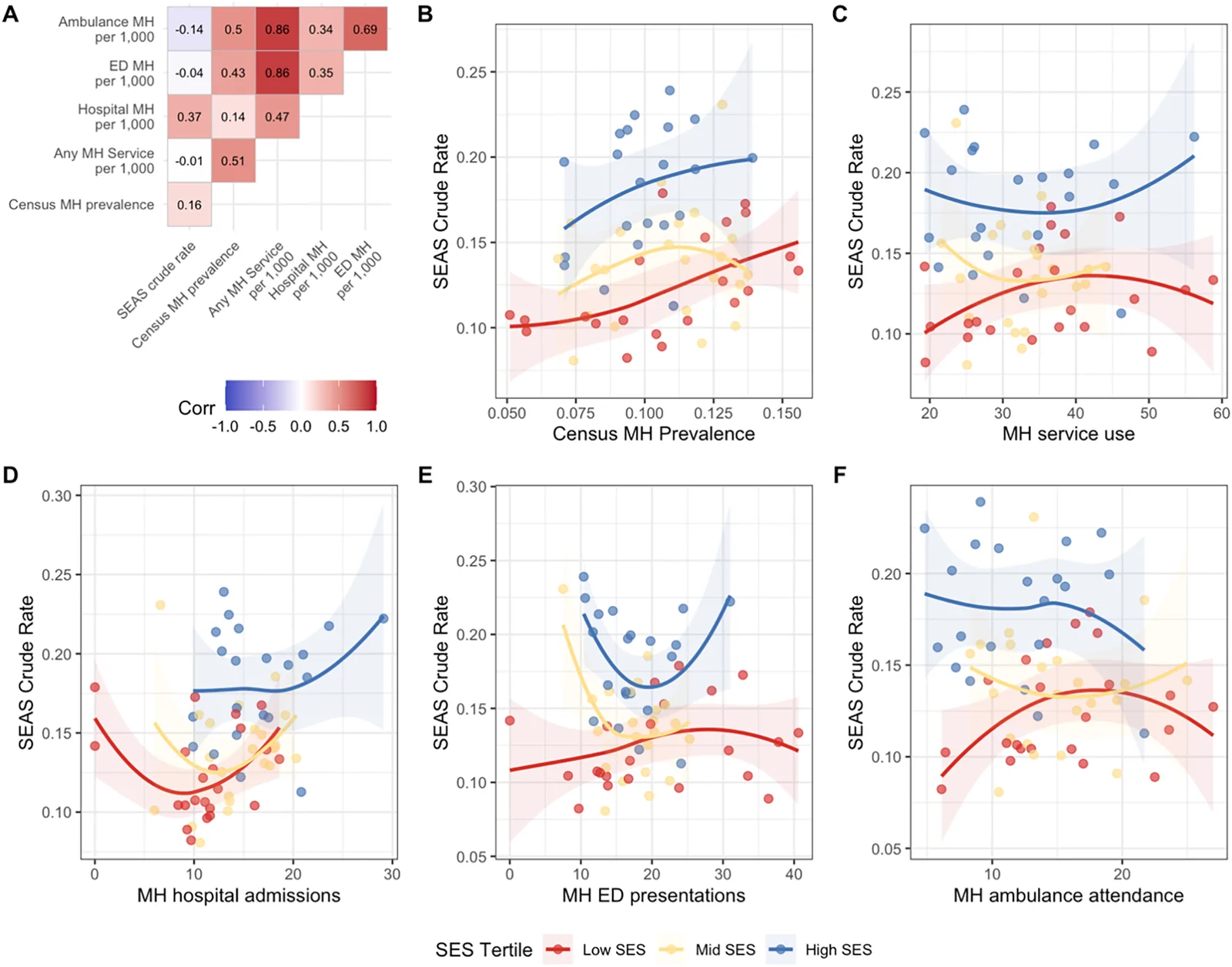
Pairwise relationships between SEAS crude rates and population indicators across Victorian SA3 regions. **(A)** Spearman correlation matrix showing pairwise associations between area-level mental health service utilisation rates (ambulance, emergency department [ED], hospital admissions, any MH service) per 1,000 population, and census-reported mental health prevalence (proportion of the population). Colour intensity reflects correlation magnitude (scale: -1.0 to 1.0). **(B–F)** Bivariate scatterplots of SEAS crude rates against **(B)** census-reported mental health prevalence, **(C)** overall MH service use, **(D)** MH hospital admissions, **(E)** MH ED presentations, and **(F)** MH ambulance attendances — with service utilisation measures expressed per 1,000 population. Points are coloured by area-level socioeconomic tertile derived from IRSAD (red = Low SES, yellow = Mid SES, blue = High SES), with LOESS smoothing curves and 95% confidence intervals shown separately for each tertile.

Census-reported mental health prevalence showed moderate positive correlations with ED presentations (r = 0.43) and ambulance attendances (r = 0.50), broadly consistent across SES tertiles, but little overall association with hospital admissions (r = 0.14), though a modest within-high-SES association was observed (r = 0.50).

In contrast, SEAS crude rates showed weak and largely non-significant correlations with all mental health indicators. The correlation with census-reported prevalence was small and non-significant overall (r = 0.16), though stronger within low-SES areas (r = 0.47). SEAS rates showed a modest positive overall correlation with hospital admissions (r = 0.37), but negligible or negative correlations with ED presentations (r = -0.04) and ambulance attendances (r = -0.14), neither of which reached significance. Within SES tertiles, correlations between SEAS rates and all service measures were weak and generally non-significant, except for a positive association with ambulance attendances in low-SES areas (r = 0.45).

### Geographic concordance and discordance

To examine alignment between mental health SEAS applications rates and population-level indicators, SA3s were independently classified into tertiles based on: SEAS rates (low < 0.13, high > 0.16), Census-reported mental health condition prevalence (low < 0.094, high: > 0.12), service utilisation data (low < 28.1, high: > 36.6 per 1,000) (**Figure 4**; **Supplementary Table 2**).

**Figure 4:**
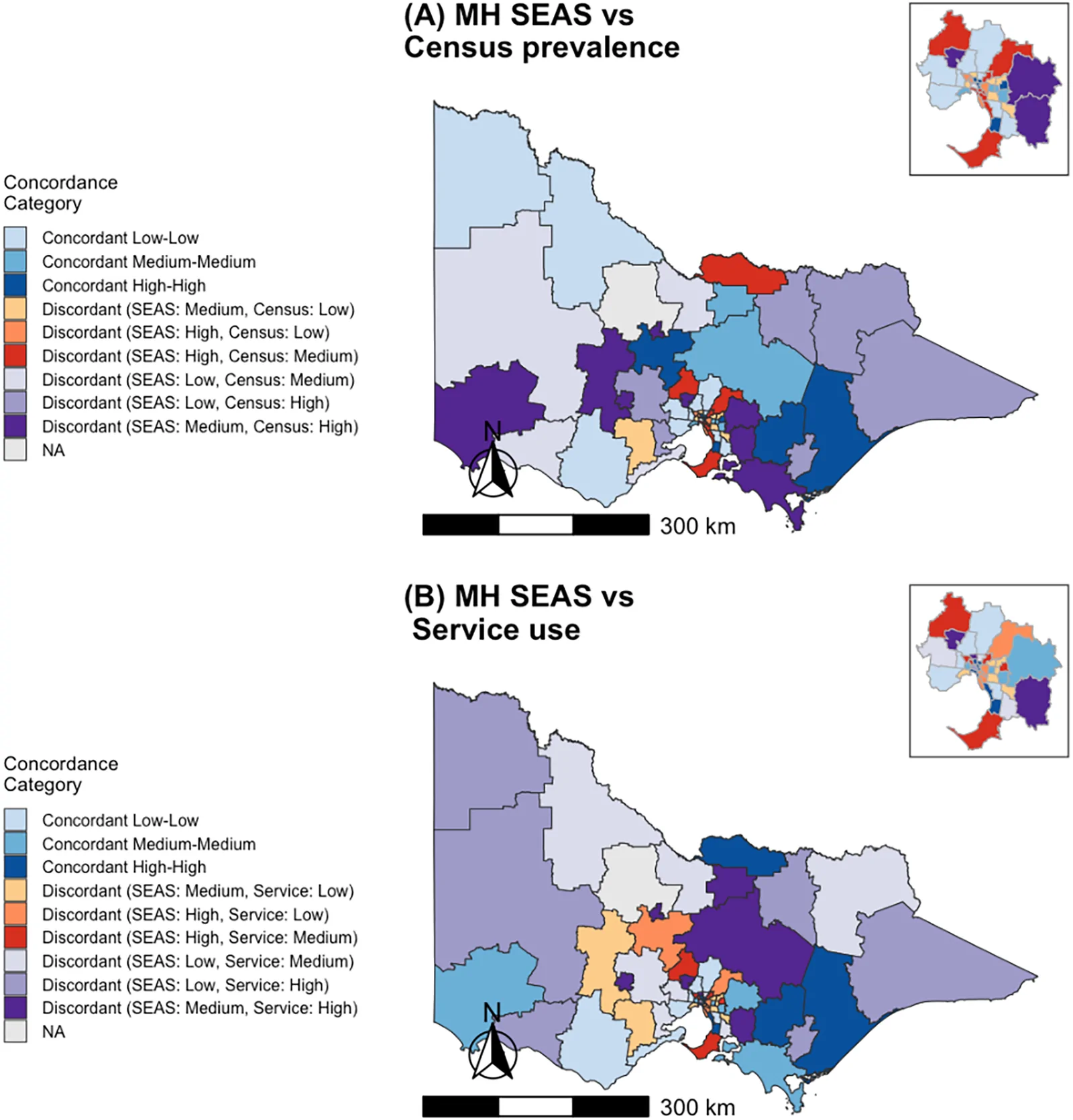
Geographic concordance between mental health-related SEAS application rates and **(A)** 2021 ABS Census-reported mental health condition prevalence, and **(B)** acute mental health service utilisation rates, across Victorian Statistical Area Level 3 (SA3) regions. SA3s are classified into tertiles (low, medium, high) on each measure. Concordant regions (matching tertile categories) are shown in blue shades; discordant regions in orange or purple shades. Insets show Greater Melbourne.

When examining concordance with Census prevalence, 25 SA3s (38%) fell within the same tertile category (11 low-low, 6 medium-medium, and 8 high-high). Of the discordant areas, 19 SA3s (29%) showed SEAS application rates in higher tertiles than their Census prevalence would suggest, while 21 SA3s (32%) showed SEAS application rates in lower tertiles than their Census prevalence. Among the extreme discordant patterns, 4 SA3s fell into the high SEAS/low Census prevalence category, exemplified by Essendon (SEAS: 0.20, Census: 0.07), suggesting potential over-representation of SEAS applications relative to population prevalence. Conversely, 6 SA3s showed the opposite pattern of low SEAS/high Census, such as Wodonga–Alpine (SEAS: 0.10, Census: 0.13), indicating potential underutilisation of SEAS pathways despite elevated mental health burden.

Concordance patterns with acute service utilisation data revealed similar overall concordance rates (22 SA3s, 34%) but important differences in geographical distribution of concordance and discordance (**Figure 4B**). Among the discordant areas, 21 SA3s (32%) showed SEAS application rates in higher tertiles than their service utilisation, while 22 SA3s (34%) showed SEAS application rates in lower tertiles than their service use. Among the extreme discordant patterns, 7 SA3s fell into the high SEAS/low Service use category, exemplified by Heathcote – Castlemaine – Kyneton (SEAS: 0.23, Service use: 6.6 per 1,000). Conversely, 7 SA3s showed the opposite pattern of low SEAS/high Service use, such as Melbourne City (SEAS: 0.11, Service use: 20.8 per 1,000).

Cross-tabulation of Census and service utilisation concordance patterns revealed 6 SA3s (Baw Baw, Brunswick – Coburg, Darebin – South, Frankston, Wellington, Yarra) that were high across all three measures, representing areas with consistently elevated mental health burden and demand. Conversely, only 5 SA3 were concordant low across all three measures, while the remaining SA3s showed varying patterns of concordance and discordance across the two measures, suggesting complex relationships between population prevalence, service engagement, and SEAS application patterns. In general, regional SA3s were more likely than metropolitan areas to show discordant patterns in which SEAS application rates fell below what Census prevalence or service utilisation rates would predict (Census: 56% of regional vs. 13% of metropolitan SA3s; service utilisation: 56% vs. 20%, respectively).

These area-level concordance and discordance classifications identify SA3 regions where aggregate SEAS application rates diverge from aggregate population-level mental health indicators and are intended to highlight communities that may warrant further investigation or targeted outreach. They cannot be used to infer that any specific individual applicant did or did not have unmet mental health need. The value of this analysis lies in identifying geographic patterns for further investigation and resource planning, rather than in drawing conclusions about individual applicants’ circumstances.

## Discussion

For young people with mental ill-health, the transition from secondary to tertiary education represents both a window of opportunity and a point of vulnerability. Therefore, understanding who reaches formal support pathways at this juncture, and who does not, is essential for equitable access to higher education. This study provides the first comprehensive characterisation of mental health-related Special Entry Access Scheme (SEAS) applications among Victorian Year-12 tertiary education applicants, integrating population-level mental health prevalence and acute service utilisation data across SA3 regions. The data showed that 15% of Victorian Year-12 VTAC applicants in the 2021/22 admissions cycle reported a mental health condition through SEAS, which represents a substantial rate and affirming the scheme’s relevance and reach within this educationally engaged cohort. This overall uptake was accompanied by marked demographic disparities. Female applicants were nearly twice as likely as males to disclose a mental health condition, and non-binary applicants were more than three times as likely as males, consistent with well-documented higher rates of mental ill-health among these groups (14, 15). For both female and non-binary applicants, these elevated rates may be shaped by experiences of stigma, discrimination, and social exclusion rather than individual characteristics alone (16). Applicants from non-English speaking backgrounds were half as likely as English speakers to apply for mental health SEAS, and those from higher socioeconomic areas and metropolitan regions showed greater uptake of the scheme, suggesting that cultural and structural barriers may contribute to who reaches this pathway beyond underlying need alone.

The socioeconomic patterning of mental health SEAS applications warrants particular attention, as the data reveal a seemingly paradoxical relationship operating simultaneously at individual and area levels, observed within this single, post-pandemic admissions cohort. At the individual level, applicants reporting financial hardship were 52% more likely to apply for mental health SEAS, consistent with a substantial body of evidence linking economic adversity to mental ill-health through pathways including chronic stress, material deprivation, and reduced access to protective resources (17). Yet at the area level, the relationship operated in the opposite direction: each decile increase in area-level socioeconomic advantage was associated with an 8% higher rate of mental health SEAS applications, with the steepest gradient concentrated among applicants from the most advantaged areas. This pattern reflects a well-recognised phenomenon in health services research whereby need and utilisation diverge across the socioeconomic spectrum, in which access to, and engagement with, health and welfare systems is shaped not only by underlying burden but by the resources, mental health literacy, and social capital required to navigate them (18). Applicants from higher socioeconomic areas may be more likely to hold a formal mental health diagnosis, given well-documented gradients in access to private mental health care and specialist services (19), as well as more likely to possess awareness of the accommodations available to them, confidence in self-advocacy, and familiarity with tertiary admission processes (20). Taken together, these findings suggest that while financial hardship may increase vulnerability to mental ill-health, community-level advantage facilitates the translation of that need into a formal application.

This dynamic is reflected in the discordance patterns observed in areas such as Wangaratta-Benalla, Geelong, Grampians, Wodonga-Alpine, Warrnambool, and Latrobe Valley which showed lower SEAS application rates relative to both Census prevalence and acute service utilisation. More broadly, regional SA3s were more likely than metropolitan areas to show discordant patterns in which SEAS application rates fell below what Census prevalence or service utilisation rates would predict (Census: 56% of regional vs. 13% of metropolitan SA3s; service utilisation: 56% vs. 20%, respectively). This pattern may reflect several non-mutually exclusive explanations: structural barries to mental health service access in regional Australia, including workforce shortages, geographic distance, and limited specialist services (21), but also differences in awareness of SEAS provisions among students, families and schools, variation in school-based navigation support, or differences in help-seeking behaviour. These patterns warrant replication beyond this single, pandemic-affected cohort before being treated as an established structural feature.

Nonetheless, the modest positive correlation between SEAS application rates and hospital admissions offers some support for the scheme reaching communities with elevated mental health need, though this relationship was not consistent across other indicators examined. Areas with consistently high rates across all three measures (Baw Baw, Brunswick–Coburg, Darebin–South, Frankston, Wellington, and Yarra) represent communities where mental health burden is high and SEAS uptake broadly aligns with that burden, though this pattern was not observed uniformly across the dataset and should be interpreted as illustrative rather than evidence that the pathway is functioning as intended more generally.

The high degree of intersectionality observed in this cohort, with 89% of mental health SEAS applicants also applying under at least one additional category, underscores that mental ill-health rarely occurs in isolation but is embedded within broader patterns of disadvantage, reflecting well-documented bidirectional relationships between social adversity and psychological distress (22). The magnitude of this co-occurrence in the present cohort is likely inflated by the temporary COVID-specific SEAS option available during this admissions cycle. However, elevated co-occurrence with financial hardship, difficult life circumstances, and physical health comorbidities was evident even considering this, consistent with evidence that poverty, housing instability, and identifying as culturally or sexually diverse confer both direct and cumulative risk for mental ill-health (23). The high co-occurrence with learning disabilities is consistent with well-documented elevated rates of anxiety and depression among young people with learning difficulties, likely reflecting the cumulative psychological toll of academic struggle and insufficient educational support (24).

This raises a broader question about the function of mental health SEAS within the tertiary admissions process that warrants further investigation. Beyond its role in adjusting entry requirements, it is possible that the inclusion of mental health conditions within the SEAS medical condition or disability category may itself serve as an access-enabling signal that legitimises disclosure and encourages young people with mental health conditions to pursue higher education who might otherwise not have considered it a viable pathway. If so, SEAS functions not merely as an equity correction mechanism but as an active enabler of participation. It also, however, carries an implicit commitment: students who disclose a mental health condition at the point of application may reasonably expect that recognition of their circumstances does not end at the offer stage. The transition into higher education is itself a well-documented risk period for mental health deterioration (25), and for students who have already identified as struggling, the adequacy of institutional support once enrolled is critical. The equity intent embedded in SEAS at the admissions stage must therefore be matched by sustained, adequately resourced mental health support throughout the higher education experience.

### Policy implications

These findings, drawn from a single post-pandemic admissions cycle, point to several areas where policy attention is warranted to improve both the equity and effectiveness of mental health SEAS as an access pathway.

#### Identify students who would benefit from wellbeing support earlier

Proactively identifying students who may benefit from additional support from career practitioners, welfare and counselling staff earlier, such as in year 11 or earlier, means that students will be better supported to address acute concerns and have an understanding of the supports available by the time they need to apply for SEAS, rather than relying on students and families to self-navigate. This is particularly important in low-income schools and regional areas where institutional support capacity is lowest and access to clinical services for documentation purposes most constrained. This support would include timely guidance on collecting information (which may require mental health service appointments and wait times) for an application, and the appropriate equity categories to apply for.

#### Reach culturally and linguistically diverse communities

Lower application rates among applicants from non-English speaking backgrounds may reflect multiple, overlapping barriers. Limited awareness of SEAS provisions and language to navigating the application process, as well as cultural stigma around mental ill-health may deter young people and their families to formally document a mental health condition. Addressing these barriers requires more than translated resources. It requires sustained, culturally safe engagement with communities through primary care, schools, and community groups, to build understanding of what SEAS is, what disclosure does and does not mean for a student’s record, and how the scheme can support rather than stigmatise. Continuing professional development for GPs and clinicians supporting SEAS documentation for CALD patients is also warranted.

#### Target areas with high crisis service use but low SEAS uptake

Geographic discordance between acute mental health service utilisation and SEAS application rates identifies specific communities where young people are accessing crisis care but not reaching the SEAS pathway. In these areas, schools and community mental health services would benefit from additional resources and awareness programs aimed at increasing mental health support and supported access to SEAS, for example ensuring that clinicians already in contact with these young people are equipped and resourced to support SEAS documentation.

### Limitations

This study has several limitations to consider when interpreting the findings. First, SEAS applications represent only a subset of young people experiencing mental ill-health. Applying for mental health SEAS requires multiple steps, including obtaining supporting documentation from a medical practitioner, being aware of the scheme, and being willing to disclose mental health conditions in an application context. Some young people with mental ill-health may not apply to university or TAFE at all, due to the severity of their condition, insufficient academic preparation, financial barriers, or other competing priorities. SEAS application rates therefore reflect the intersection of mental health prevalence, educational aspiration, service access, and disclosure comfort, rather than mental health need alone.

Second, this study used a cross-sectional design examining a single application cohort, which prevents any causal conclusions from being drawn. The geographic analysis examines area-level associations that may not reflect individual-level relationships due to ecological fallacy. We cannot determine whether the spatial variation in SEAS application rates reflects true differences in mental health prevalence, differences in the proportion of young people seeking tertiary education, variation in awareness of SEAS, or differences in service access and diagnostic practices across regions.

Third, this study only captures young people who apply to university through VTAC, representing a selected subset of the youth population. It excludes those who did not pursue tertiary education at all, those who left secondary school early, or followed vocational or non-ATAR pathways (e.g., Unscored VCE or VCE Vocational Major). These excluded groups may differ systematically in mental health profile and may include those with the greatest unmet need. Consequently, young people whose mental health significantly impairs their educational attainment may be underrepresented, potentially underestimating the true burden of mental health challenges among young Victorians. Furthermore, the decision to disclose mental health conditions in SEAS applications is voluntary and may be influenced by stigma, perceived benefits, previous experiences with disclosure, and cultural factors, introducing additional selection bias.

Fourth, we were unable to account for several contextual factors that likely influence both mental health and SEAS application patterns. School-level factors such as counselling resources, teacher awareness of SEAS, and institutional culture around mental health disclosure may significantly affect application rates. The quality, accessibility, and diagnostic practices of mental health services vary across regions, potentially contributing to geographic variation in SEAS applications independent of true prevalence differences. These unmeasured factors limit our ability to fully explain the patterns observed. Additionally, our approach to estimating Year-12-equivalent population denominators (halving Census-reported counts of 17–18-year-olds) assumes stable population size across adjacent single-year ages within each SA3; this assumption was not formally tested via sensitivity analysis using alternative denominators, and SA3s with atypical age-structure (e.g., due to localised migration patterns) may be more susceptible to denominator misestimation than assumed under a stable-population model.

Fifth, and critically, SEAS application data capture only the point of entry into tertiary education. Whether students who disclosed mental ill-health at application subsequently enrolled, persisted, and completed their studies remains unknown. Given that the transition into tertiary education is a well-documented risk period for mental health deterioration, students with pre-existing mental ill-health may be particularly vulnerable to attrition. Longitudinal data linking admissions records to enrolment, academic progression, and completion outcomes across both universities and TAFEs are needed to assess whether special consideration at the point of application translates into equitable educational outcomes over time.

Finally, our findings are specific to Victoria, Australia, and may not generalise to other jurisdictions with different educational systems, SEAS policies, or mental health service landscapes. The single-year cross-sectional design also limits our understanding of temporal trends; SEAS application patterns may have been affected by the COVID-19 pandemic, which substantially impacted youth mental health and educational experiences during the years preceding this application cohort. Ongoing monitoring of population trends is warranted to inform policy, service implementation and strengthen the capacity of mental health and education systems to address young people’s needs and broader social determinants that shape them.

Despite these limitations, our findings provide important population-level insights into the intersection of mental health, educational disadvantage, and university access among young Victorians. The patterns observed have implications for equity in higher education access and suggest directions for targeted intervention and support.

## Data Availability

The data analysed in this study is subject to the following licenses/restrictions: Data used in this paper can be requested from VTAC and the corresponding author, subject to approval by VTAC, compliance with relevant privacy and ethical requirements, and execution of any required data access agreements. Due to the sensitive nature of the data and privacy obligations to participants, the dataset is not publicly available. Requests to access these datasets should be directed to carolineg@unimelb.edu.au.
