# Supplementary material for: Mental health special consideration in Victorian tertiary education applications: geographical, demographic, and socioeconomic inequities in uptake
Source: Front Psychiatry. 2026 Sep 15;17:1908088. doi: 10.3389/fpsyt.2026.1908088 (PMC13619618; doi:10.3389/fpsyt.2026.1908088)
Supplement: Supplementary file 1 [file SupplementaryFile1.docx]

Supplementary Table 1: Participant characteristics of all VTAC applications from the 2021/22 admissions cycle

|  | **Total (N=62655)** | **Older Non-Victorian (N=1807)** | **Older Victorian (N=11475)** | **Year 12 Non-Victorian (N=6246)** | **Year 12 Victorian (N=43127)** |
| --- | --- | --- | --- | --- | --- |
| **Gender** |  |  |  |  |  |
| Male | 25425 (40.6%) | 717 (39.7%) | 3764 (32.8%) | 2238 (35.8%) | 18706 (43.4%) |
| Female | 36789 (58.7%) | 1068 (59.1%) | 7560 (65.9%) | 3972 (63.6%) | 24189 (56.1%) |
| Non-binary | 441 (0.7%) | 22 (1.2%) | 151 (1.3%) | 36 (0.6%) | 232 (0.5%) |
| **Born overseas** | 13888 (22.2%) | 482 (26.7%) | 3086 (26.9%) | 1929 (30.9%) | 8391 (19.5%) |
| **Language other than English** | 11375 (18.2%) | 318 (17.6%) | 2148 (18.7%) | 1445 (23.1%) | 7464 (17.3%) |
| **Aboriginal and/or Torres Strait Islander** | 467 (0.7%) | 26 (1.4%) | 74 (0.6%) | 87 (1.4%) | 280 (0.6%) |
| **Parent university education** |  |  |  |  |  |
| None | 17493 (28.0%) | 413 (23.1%) | 4183 (37.0%) | 781 (12.5%) | 12116 (28.1%) |
| One | 14945 (23.9%) | 416 (23.3%) | 2784 (24.6%) | 1131 (18.1%) | 10614 (24.6%) |
| Both | 29995 (48.0%) | 960 (53.7%) | 4348 (38.4%) | 4330 (69.4%) | 20357 (47.2%) |
| **First family member attending university** | 9602 (15.3%) | 79 (4.4%) | 1011 (8.8%) | 314 (5.0%) | 8198 (19.0%) |
| **IRSAD percentile** |  |  |  |  |  |
| Mean (SD) | 60.4 (28.3) | 61.3 (30.6) | 60.5 (27.9) | 63.9 (30.4) | 59.8 (27.9) |
| Median (Q1, Q3) | 63.0 (38.0, 86.0) | 67.0 (34.0, 91.0) | 63.0 (39.0, 86.0) | 72.0 (36.0, 93.0) | 62.0 (39.0, 86.0) |
| **ATAR** |  |  |  |  |  |
| <50 | 7836 (13.6%) | 98 (6.6%) | 1640 (17.8%) | 161 (2.7%) | 5937 (14.6%) |
| (50,70] | 14832 (25.8%) | 241 (16.1%) | 2326 (25.3%) | 698 (11.5%) | 11567 (28.5%) |
| (70,80] | 9714 (16.9%) | 226 (15.1%) | 1518 (16.5%) | 747 (12.3%) | 7223 (17.8%) |
| (80,90] | 11066 (19.3%) | 408 (27.3%) | 1807 (19.6%) | 1167 (19.2%) | 7684 (18.9%) |
| (90,95] | 6173 (10.8%) | 241 (16.1%) | 997 (10.8%) | 968 (16.0%) | 3967 (9.8%) |
| >95 | 7789 (13.6%) | 281 (18.8%) | 915 (9.9%) | 2324 (38.3%) | 4269 (10.5%) |
| **Special consideration for mental health** | 7155 (11.4%) | 49 (2.7%) | 475 (4.1%) | 284 (4.5%) | 6347 (14.7%) |
|  |  |  |  |  |  |
|  |  |  |  |  |  |
| **Special consideration for financial hardship** | 8519 (13.6%) | 95 (5.3%) | 993 (8.7%) | 382 (6.1%) | 7049 (16.3%) |
| **Special consideration for difficult life or family challenges** | 32371 (51.7%) | 86 (4.8%) | 1227 (10.7%) | 747 (12.0%) | 30311 (70.3%) |
| **Medical condition** | 1513 (2.4%) | 27 (1.5%) | 249 (2.2%) | 69 (1.1%) | 1168 (2.7%) |
| **COVID Impact** | 30189 (48.2%) | 9 (0.5%) | 570 (5.0%) | 509 (8.1%) | 29101 (67.5%) |

Supplementary Table 2: SEAS rates, census prevalence, and acute service utilisation rates and tertiles for 65 SA3 regions

| SA3 Name | SEAS Crude Rate | SEAS per 1,000 | Census MH Prevalence | Census population count | Hosp. MH Rate per 1,000 | Ambulance MH Rate per 1,000 | ED MH Rate per 1,000 | Any MH Service Rate per 1,000 | SEAS Tertile | Census Tertile | Service Tertile | Hospital Tertile | Ambulance Tertile | ED Tertile |
| --- | --- | --- | --- | --- | --- | --- | --- | --- | --- | --- | --- | --- | --- | --- |
| Ballarat | 0.13 | 47.72 | 0.14 | 1404 | 17.1 | 19.6 | 17.8 | 41.3 | Medium | High | High | High | High | Medium |
| Banyule | 0.2 | 145.63 | 0.11 | 1339 | 14.2 | 12.7 | 19.8 | 32.1 | High | Medium | Medium | Medium | Medium | Medium |
| Barwon - West | 0.16 | 66.33 | 0.09 | 301.5 | 11.6 | 8.3 | 11.6 | 21.6 | Medium | Low | Low | Medium | Low | Low |
| Baw Baw | 0.17 | 74.88 | 0.14 | 641 | 10.1 | 16.4 | 32.8 | 46 | High | High | High | Low | High | High |
| Bayside | 0.21 | 166.61 | 0.09 | 1434.5 | 12.2 | 10.5 | 12.5 | 25.8 | High | Low | Low | Medium | Low | Low |
| Bendigo | 0.13 | 58.43 | 0.16 | 1181 | 18.6 | 23.7 | 40.6 | 58.8 | Medium | High | High | High | High | High |
| Boroondara | 0.2 | 179.35 | 0.09 | 2392 | 12.8 | 6.9 | 11.7 | 23 | High | Low | Low | Medium | Low | Low |
| Brimbank | 0.1 | 47.3 | 0.06 | 2135.5 | 8.4 | 12.2 | 8.2 | 20.1 | Low | Low | Low | Low | Medium | Low |
| Brunswick - Coburg | 0.19 | 135.51 | 0.12 | 642 | 19.5 | 15.6 | 23.4 | 45.2 | High | High | High | High | Medium | High |
| Campaspe | 0.1 | 40.77 | 0.1 | 441.5 | 11.3 | 17 | 23.8 | 34 | Low | Medium | Medium | Low | High | High |
| Cardinia | 0.13 | 57.62 | 0.13 | 1423 | 17.6 | 17.6 | 25.3 | 40.1 | Medium | High | High | High | High | High |
| Casey - North | 0.13 | 103.19 | 0.08 | 1773.5 | 11.6 | 10.1 | 12.4 | 24.2 | Medium | Low | Low | Medium | Low | Low |
| Casey - South | 0.11 | 50.55 | 0.08 | 2750 | 13.6 | 12.7 | 19.1 | 30.7 | Low | Low | Medium | Medium | Medium | Medium |
| Colac - Corangamite | 0.08 | 31.59 | 0.09 | 411.5 | 9.7 | 6.1 | 9.7 | 19.4 | Low | Medium | Low | Low | Low | Low |
| Creswick - Daylesford - Ballan | 0.09 | 29.36 | 0.12 | 306.5 | 9.8 | 19.6 | 19.6 | 32.6 | Low | High | Medium | Low | High | Medium |
| Dandenong | 0.1 | 55.15 | 0.06 | 2103.5 | 11.6 | 11.4 | 13.8 | 25.2 | Low | Low | Low | Medium | Medium | Low |
| Darebin - North | 0.12 | 95.29 | 0.09 | 850 | 14.7 | 13.5 | 18.2 | 32.9 | Low | Low | Medium | Medium | Medium | Medium |
| Darebin - South | 0.2 | 162.16 | 0.14 | 499.5 | 21 | 19 | 17 | 39 | High | High | High | High | High | Medium |
| Essendon | 0.2 | 209.18 | 0.07 | 664.5 | 17.3 | 15 | 16.6 | 35.4 | High | Low | Medium | High | Medium | Medium |
| Frankston | 0.17 | 63.9 | 0.14 | 1518 | 16.8 | 18.1 | 20.4 | 36.6 | High | High | High | High | High | High |
| Geelong | 0.13 | 58.31 | 0.13 | 2418 | 12.8 | 16.5 | 19.4 | 35.4 | Low | High | Medium | Medium | High | Medium |
| Gippsland - East | 0.13 | 45.31 | 0.13 | 463.5 | 11.9 | 27 | 37.8 | 55 | Medium | High | High | Medium | High | High |
| Gippsland - South West | 0.15 | 72.88 | 0.12 | 713.5 | 14.7 | 12.6 | 21.7 | 35 | Medium | High | Medium | Medium | Medium | High |
| Glen Eira | 0.22 | 197.85 | 0.09 | 1723.5 | 14.5 | 8.7 | 14.5 | 26.1 | High | Medium | Low | Medium | Low | Low |
| Glenelg - Southern Grampians | 0.14 | 54.86 | 0.13 | 437.5 | 9.1 | 13.7 | 13.7 | 32 | Medium | High | Medium | Low | Medium | Low |
| Grampians | 0.1 | 40.13 | 0.12 | 623 | 16.1 | 16.1 | 13.6 | 37.7 | Low | Medium | High | High | High | Low |
| Heathcote - Castlemaine - Kyneton | 0.23 | 67.86 | 0.13 | 530.5 | 6.6 | 13.2 | 7.5 | 23.6 | High | High | Low | Low | Medium | Low |
| Hobsons Bay | 0.16 | 86.55 | 0.11 | 855 | 9.9 | 9.9 | 17 | 26.3 | Medium | Medium | Low | Low | Low | Medium |
| Keilor | 0.16 | 128.49 | 0.07 | 716 | 10.5 | 9.1 | 14 | 28.6 | High | Low | Medium | Low | Low | Low |
| Kingston | 0.19 | 177.6 | 0.1 | 1357 | 21.4 | 14 | 22.8 | 39.1 | High | Medium | High | High | Medium | High |
| Knox | 0.15 | 97.61 | 0.11 | 1844 | 16.8 | 13.8 | 20.6 | 34.7 | Medium | Medium | Medium | High | Medium | High |
| Latrobe Valley | 0.11 | 35.98 | 0.13 | 889.5 | 12.4 | 23.6 | 16.9 | 39.3 | Low | High | High | Medium | High | Medium |
| Macedon Ranges | 0.19 | 145.95 | 0.11 | 438.5 | 18.2 | 21.7 | 19.4 | 35.3 | High | Medium | Medium | High | High | Medium |
| Manningham - East | 0.16 | 98.13 | 0.09 | 428 | 17.5 | 5.8 | 16.4 | 19.9 | Medium | Low | Low | High | Low | Medium |
| Manningham - West | 0.14 | 112.04 | 0.07 | 1080 | 12 | 12.5 | 15.3 | 25.9 | Medium | Low | Low | Medium | Medium | Medium |
| Maribyrnong | 0.13 | 78.02 | 0.09 | 666.5 | 20.3 | 15 | 20.3 | 34.5 | Medium | Low | Medium | High | Medium | High |
| Maroondah | 0.17 | 97.73 | 0.12 | 1279 | 14.5 | 11.3 | 18 | 29.7 | High | High | Medium | Medium | Low | Medium |
| Maryborough - Pyrenees | 0.14 | 147 | 0.15 | 258.5 | 0 | 9.7 | 0 | 19.3 | Medium | High | Low | Low | Low | Low |
| Melbourne City | 0.11 | 35.87 | 0.11 | 1059.5 | 20.8 | 21.7 | 24.1 | 46.2 | Low | Medium | High | High | High | High |
| Melton - Bacchus Marsh | 0.1 | 68.03 | 0.09 | 2543 | 13.4 | 15.3 | 15.9 | 31.7 | Low | Low | Medium | Medium | Medium | Medium |
| Mildura | 0.1 | 36.59 | 0.09 | 656 | 9.1 | 13 | 33.5 | 41.2 | Low | Low | High | Low | Medium | High |
| Moira | 0.18 | 69.95 | 0.11 | 314.5 | 0 | 17.5 | 23.8 | 36.6 | High | Medium | High | Low | High | High |
| Monash | 0.14 | 98.68 | 0.07 | 2118 | 9.9 | 8.3 | 11.8 | 21.2 | Medium | Low | Low | Low | Low | Low |
| Moreland - North | 0.14 | 95.24 | 0.07 | 693 | 18 | 16.6 | 17.3 | 36.8 | Medium | Low | High | High | High | Medium |
| Mornington Peninsula | 0.16 | 85.17 | 0.11 | 1878.5 | 19.2 | 11.4 | 17 | 33.3 | High | Medium | Medium | High | Medium | Medium |
| Murray River - Swan Hill | 0.1 | 43.7 | 0.08 | 389 | 11.6 | 6.4 | 16.7 | 28.3 | Low | Low | Medium | Medium | Low | Medium |
| Nillumbik - Kinglake | 0.17 | 123.41 | 0.11 | 980.5 | 14.3 | 7.6 | 13.8 | 27 | High | Medium | Low | Medium | Low | Low |
| Port Phillip | 0.22 | 202.83 | 0.11 | 636 | 23.6 | 15.7 | 24.4 | 42.5 | High | Medium | High | High | Medium | High |
| Shepparton | 0.14 | 57.63 | 0.11 | 815.5 | 15.9 | 20.2 | 23.3 | 41.7 | Medium | Medium | High | Medium | High | High |
| Stonnington - East | 0.22 | 179.19 | 0.1 | 519 | 13.5 | 4.8 | 10.6 | 19.3 | High | Medium | Low | Medium | Low | Low |
| Stonnington - West | 0.24 | 212.99 | 0.11 | 385 | 13 | 9.1 | 10.4 | 24.7 | High | Medium | Low | Medium | Low | Low |
| Sunbury | 0.14 | 65.26 | 0.13 | 521 | 18.2 | 25 | 20.2 | 44.1 | Medium | High | High | High | High | Medium |
| Surf Coast - Bellarine Peninsula | 0.11 | 60.54 | 0.12 | 925 | 13.5 | 11.4 | 13.5 | 25.4 | Low | Medium | Low | Medium | Medium | Low |
| Tullamarine - Broadmeadows | 0.11 | 65.07 | 0.05 | 2520.5 | 10.1 | 11.1 | 12.3 | 26.4 | Low | Low | Low | Low | Low | Low |
| Upper Goulburn Valley | 0.14 | 57.71 | 0.1 | 606.5 | 17.3 | 19 | 19.8 | 37.1 | Medium | Medium | High | High | High | Medium |
| Wangaratta - Benalla | 0.12 | 48.87 | 0.14 | 552.5 | 10.9 | 17.2 | 30.8 | 48 | Low | High | High | Low | High | High |
| Warrnambool | 0.09 | 38.76 | 0.11 | 645 | 9.3 | 22.5 | 36.4 | 50.4 | Low | Medium | High | Low | High | High |
| Wellington | 0.16 | 58.76 | 0.13 | 493.5 | 14.2 | 14.2 | 28.4 | 38.5 | High | High | High | Medium | Medium | High |
| Whitehorse - East | 0.15 | 114.7 | 0.1 | 697.5 | 14.3 | 7.2 | 19.4 | 28 | Medium | Medium | Medium | Medium | Low | Medium |
| Whitehorse - West | 0.16 | 130.86 | 0.1 | 1291.5 | 17 | 13.6 | 16.3 | 34.8 | Medium | Medium | Medium | High | Medium | Medium |
| Whittlesea - Wallan | 0.08 | 47.09 | 0.07 | 2867 | 10.6 | 10.5 | 13.4 | 25.1 | Low | Low | Low | Low | Low | Low |
| Wodonga - Alpine | 0.1 | 30.77 | 0.13 | 910 | 6 | 13.2 | 22.5 | 33 | Low | High | Medium | Low | Medium | High |
| Wyndham | 0.11 | 74.06 | 0.08 | 3119 | 11.1 | 11.9 | 12.7 | 25.3 | Low | Low | Low | Low | Medium | Low |
| Yarra | 0.22 | 166.67 | 0.12 | 516 | 29.1 | 18.4 | 31 | 56.2 | High | High | High | High | High | High |
| Yarra Ranges | 0.15 | 79.58 | 0.13 | 1897.5 | 16.1 | 15 | 21.9 | 34.3 | Medium | High | Medium | High | Medium | High |

**Supplementary Table S3:** Sensitivity analysis – univariate and multivariable relative risk regression models examining factors associated with mental health SEAS applications among Victorian Year-12 VTAC applicants, excluding mental health SEAS applications without documented supporting evidence, 2021/22 admissions cycle. Corresponds to Table 2 in the main manuscript.

|  | **Univariate** | | **Multivariable** | |
| --- | --- | --- | --- | --- |
| **Predictor** | **RR (95% CI)** | **p-value** | **RR (95% CI)** | **p-value** |
| **Gender (Ref: Male)** |  |  |  |  |
| Female | 2.24 (2.11 - 2.38) | <0.001 | 2.26 (2.14 - 2.39) | <0.001 |
| Non-binary | 4.60 (3.68 - 5.76) | <0.001 | 4.13 (3.26 - 5.24) | <0.001 |
| **Learning disability** | 3.71 (3.30 - 4.17) | <0.001 | 3.19 (2.83 - 3.60) | <0.001 |
| **Home language – English (Ref: Other)** | 2.09 (1.86 - 2.36) | <0.001 | 2.05 (1.82 - 2.32) | <0.001 |
| **Physical condition** | 1.89 (1.58 - 2.25) | <0.001 | 1.76 (1.47 - 2.11) | <0.001 |
| **Special consideration for financial hardship** | 1.29 (1.17 - 1.42) | <0.001 | 1.54 (1.42 - 1.67) | <0.001 |
| **Greater Melbourne (Ref: Rest of Vic)** | 1.23 (1.08 - 1.41) | 0.002 | 1.09 (0.96 - 1.25) | 0.185 |
| **IRSAD decile*** | 1.09 (1.07 - 1.10) | <0.001 | 1.09 (1.07 - 1.11) | <0.001 |
| **Aboriginal and/or Torres Strait Islander** | 1.22 (0.89 - 1.66) | 0.213 | 1.10 (0.81 - 1.49) | 0.552 |
| **First family member attending university** | 0.96 (0.87 - 1.05) | 0.353 | 0.95 (0.89 - 1.03) | 0.209 |

Supplementary Table S4: SES-tertile-stratified correlations between mental health-related SEAS crude rates, census-reported mental health prevalence, and acute mental health service utilisation across Victorian SA3 regions

| **Var1** | **Var2** | **Low SES** | **Mid SES** | **High SES** |
| --- | --- | --- | --- | --- |
| **Any MH Service**  **per 1,000** | Ambulance MH  per 1,000 | 0.81 | 0.86 | 0.89 |
| **Any MH Service**  **per 1,000** | ED MH  per 1,000 | 0.88 | 0.74 | 0.85 |
| **Any MH Service**  **per 1,000** | Hospital MH  per 1,000 | 0.46 | 0.48 | 0.74 |
| **Census MH prevalence** | Ambulance MH  per 1,000 | 0.54 | 0.3 | 0.43 |
| **Census MH prevalence** | Any MH Service  per 1,000 | 0.53 | 0.26 | 0.51 |
| **Census MH prevalence** | ED MH  per 1,000 | 0.43 | 0.31 | 0.45 |
| **Census MH prevalence** | Hospital MH  per 1,000 | 0.24 | -0.1 | 0.52 |
| **ED MH**  **per 1,000** | Ambulance MH  per 1,000 | 0.69 | 0.63 | 0.72 |
| **Hospital MH**  **per 1,000** | Ambulance MH  per 1,000 | 0.49 | 0.29 | 0.64 |
| **Hospital MH**  **per 1,000** | ED MH  per 1,000 | 0.35 | 0.2 | 0.72 |
| **SEAS crude rate** | Ambulance MH  per 1,000 | 0.44 | -0.12 | -0.01 |
| **SEAS crude rate** | Any MH Service  per 1,000 | 0.35 | -0.08 | -0.05 |
| **SEAS crude rate** | Census MH prevalence | 0.59 | 0.12 | 0.28 |
| **SEAS crude rate** | ED MH  per 1,000 | 0.34 | -0.2 | -0.2 |
| **SEAS crude rate** | Hospital MH  per 1,000 | 0.07 | 0.44 | 0.17 |
